# Tosyl-carrageenan/alginate composite adsorbent for removal of Pb^2+^ ions from aqueous solutions

**DOI:** 10.1186/s13065-023-01103-0

**Published:** 2024-01-06

**Authors:** Mohamed Awed, Riham R. Mohamed, Kholod H. Kamal, Magdy W. Sabaa, Korany A. Ali

**Affiliations:** 1 Center of Excellence for Advanced Science, Advanced Materials and Nanotechnology Group National Research Centre, Dokki, Giza, 12622 Egypt; 2 Chemistry Department, Faculty of Science, Cairo University, Giza, Egypt; 3 Water Pollution Research Department, National Research Centre, Giza, 12622 Egypt; 4 Applied Organic Chemistry Department, National Research Centre, Dokki, Giza, 12622 Egypt

**Keywords:** Tosylation, κ-carrageenan, Na-Alginate, Hydrogel beads, Pb^2+^ ions, Adsorption

## Abstract

The current study effectively designed novel cross-linked tosyl-carrageenan/alginate (Ts-Car/Alg) beads to remove Pb^2+^ ions from their aqueous solutions. To confirm the structure of the produced matrix, characterization methods such as XRD, SEM, FTIR, and EDX were used. Batch experiments were employed in order to further evaluate the adsorption efficiency of Pb^2+^ ions. Additionally, various variables, including contact time, solution pH, adsorbent dosage, and initial concentration of Pb^2+^ ions were investigated using atomic absorption. The results of this study showed that the adsorption equilibrium increased as Pb^2+^ ions concentration increased at pH = 5.3 after a contact time of 120 min, with 0.3 g of Ts-Car/Alg that having the best adsorption capacity at 74 mg/g. The adsorption progression was further examined using the kinetic and isothermal models. With a correlation coefficient of 0.975, the Freundlich model was thought to better fit Pb^2+^ ions adsorption from the isotherm investigation. Also, the adsorption kinetics were investigated using a pseudo-second-order model with 1/n ratio of 0.683. This Ts-Car/Alg adsorbent is regarded as an effective candidate to be used for water treatment because the reusability process of produced beads was successfully completed twice, and the adsorbent maintained its ability to remove Pb^2+^ ions. The prepared Ts-Car/Alg beads are therefore excellent candidates to be used as potent Pb^2+^ ions adsorbents from their aqueous solutions. The Ts-Car/Alg beads' regeneration and reusability investigation for the removal of heavy metal ions was completed in at least two successful cycles.

## Introduction

Due to the growing populations and increased economic activity, heavy metal ions water contamination is a significant environmental issue in the modern era. Industrial processes, including mining, electroplating, leather tanning, painting, textile dyeing, and electroplating, discharged such metal ions. Pb^2+^ ions are one of the types of heavy metal ions that can deposit and accumulate in food, soil, and eventually living things [1–3]. Even at low concentrations, Pb^2+^ ions are deadly, very persistent, and extremely toxic to organisms, leading to a variety of health issues including disorders of the muscles, kidneys, liver, and brain where the lead level recommended by the Environmental Protection Agency (USEPA, 2009) is 5 μg/L [4, 5]. As a result, the removal of Pb^2+^ ions from aqueous solutions are turning into a severe problem that needs to be investigated.

Precipitation [6], oxidation [7], chemical reduction [8], ion exchanging [9], filtration [10], reverse osmosis [11], electroplating [12], flocculation [13], coagulation [13], and adsorption [14] are few methods that have been utilized to remove Pb^2+^ ions from aquatic environments. Adsorption is often regarded as one of the most advantageous techniques for managing effluents containing heavy metal ions due to its uncomplicated design, lack of secondary contamination, cheap cost, efficiency, and ease of handling [15, 16].

Several adsorbents have been used in the adsorption process such as bio-polymeric adsorbents that have received better attention in the adsorption process because they are biodegradable, inherently non-toxic, selective, effective, affordable, and environmentally acceptable [1–6]. Alginate, *K*-carrageenan, chitosan, and cellulose have attracted attention recently because of how well they work as water treatment materials [17, 18].

An excellent naturally occurring anionic polysaccharide derived from brown algae, sodium alginate (SA) mostly comprises -d-mannurinate (M) and -l-guluronate. It is biodegradable, biocompatible, and non-toxic. These groups are excellent candidates for coordination as they have active sites for chelation and removal of multivalent metal ions from their aqueous solutions due to the polymeric chains predominant (–COOH) and (–OH) groups. Moreover, sodium alginate structure alteration might be conducted to improve its functionality [19, 20].

Carrageenan is a naturally occurring, linear, sulfated, hydrophilic, and negatively charged polysaccharide that is made up of four connected 3,6-anhydrous-d-galactose (D-unit) and three linked-d-galactose (G-unit) units. Diverse types of red algae that are present in marine habitats are used to make carrageenan. Carrageenan can be divided into three major types based on the level of sulfation: kappa-, iota-, and lambda-carrageenan. All forms of carrageenan have anionic half-ester sulphate groups, which are responsible for their chemical reactivity. Iota-carrageenan has two sulphate groups per monomer compared to lambda-single carrageenan’s sulphate group per monomer [21–23]. Kappa-carrageenan only includes one sulphate group per monomeric unit. Carrageenan-based bio-composites were created and utilized for environmental purposes [24–26].

As a result of the functional groups on the carrageenan scaffold, carrageenan has used in water treatment in previous studies, and different matrices of carrageenan with other polymers or nanomaterials gave superior results in removing pollutants from water [21, 25]. The goal of the current study is to create an innovative, priced, and environmentally friendly tosyl-carrageenan/alginate (Ts-Car/Alg) adsorbent for removing Pb^2+^ ions from its aqueous solutions. Furthermore, FTIR, SEM, XRD, and EDX techniques applied to establish the produced matrix's structural integrity. Also, Pb^2+^ ions solution batch adsorption under a variety of conditions, including contact time, solution pH, adsorbent dosage, and initial concentration of Pb^2+^ ions have been conducted. In addition, this adsorbent regeneration was explored, and two kinetics namely, pseudo-first order and pseudo-second order, and two isotherm models namely, Freundlich and Langmuir models were looked at for an explanation of the adsorption mechanism of Pb^2+^ ions after regeneration.

## Materials and methods

### Materials

Materials were of analytical grade; Sodium alginate was purchased from Fisher scientific Co. (UK), viscosity 1%at 25 ^o^C: 5–40 cps. *p*-Toluene sulfonyl chloride, anhydrous lithium chloride (LiCl), *N, n*-dimethyl acetamide (DMA), triethyl amine (TEA), and carrageenan from Sigma-Aldrich Co. The following products: ethanol (99%), calcium chloride (CaCl_2_), sodium hydroxide (NaOH), and hydrochloric acid (HCl) from ELNASER Co. (Egypt).

### Methods

#### Adsorbent synthesis

##### Preparation of Tosyl k-carrageenan (Ts-Car)

Tosyl k-carrageenan (Ts-Car) was prepared using a previously reported approach [27, 28], with a minor modification. Briefly, a mixture of 3.0 g of k-carrageenan in 100 mL DMA was stirred and heated for 10 h at 80 °C under reflux. The carrageenan solution continuously stirred with the addition of a solution of 5 g of anhydrous LiCl in 25 mL DMA, left to cool in room temperature and then agitated for 6 h (partially dissolution). A diluted solution of TEA and DMA (10 mL: 25 mL) was added while stirring at low temperature (0–8 °C). After that, 6 g of *p-*toluene sulfonyl chloride in 25 mL DMA was added drop-by-drop to the mixture while stirring for 45 min. The reaction mixture was stirred for six hours at 0–8 °C then progressively added to 0.5 L ethanol with stirring for 15 min. Filtration and washing with ethanol followed by drying at 50 °C for 1 h in an oven was used to get precipitation of Ts-Car.

##### Preparation of Tosyl *k*-carrageenan/ Alginate (Ts-Car/Alg) beads

**Fig. 1:**
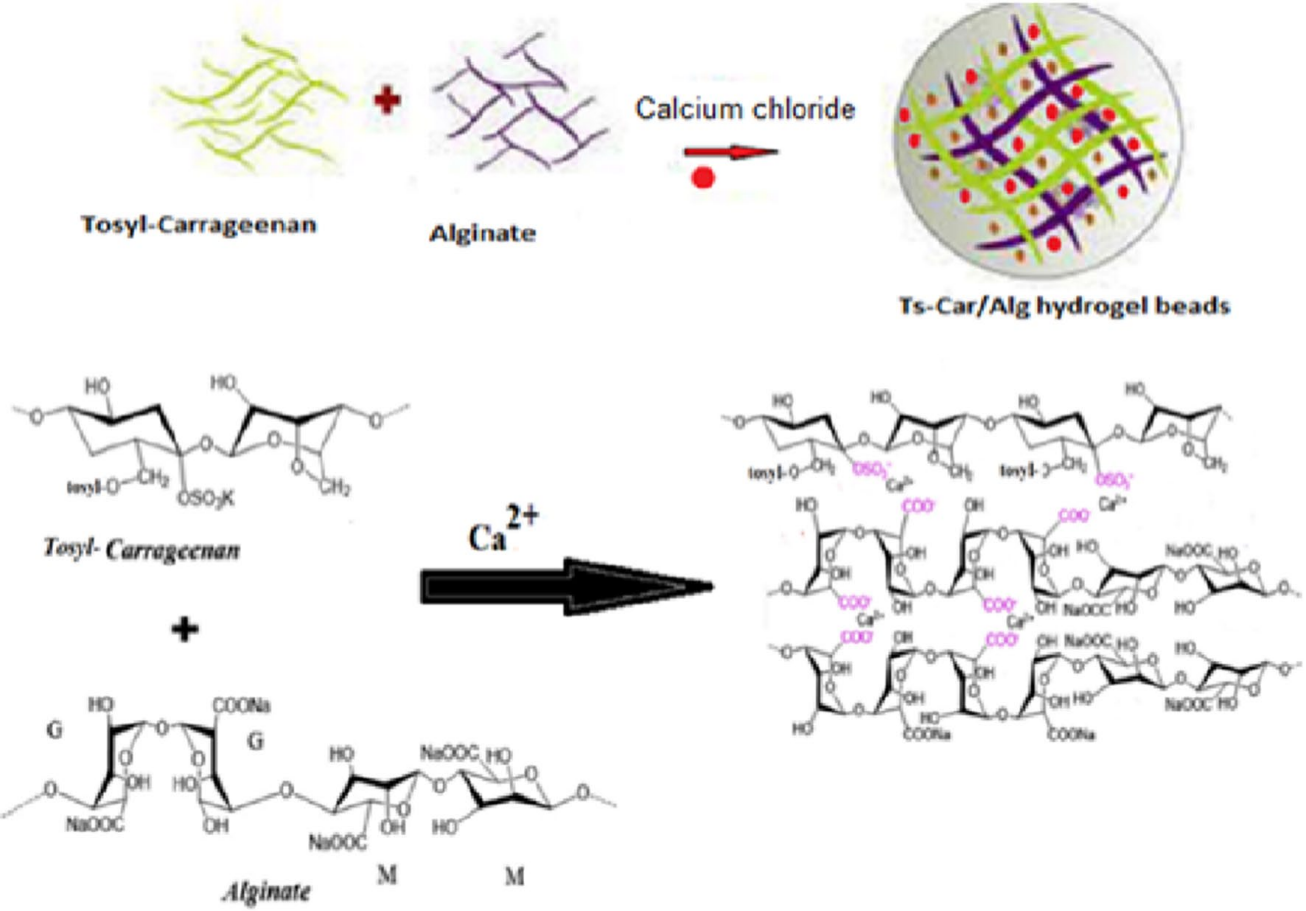
Schematic diagram of Tosyl-carrageenan/Alginate (Ts-Car/Alg) beads preparation

According to a previously documented methodology [29, 30], with a slight modification. Tosyl k-carrageenan/Alginate (Ts-Car/Alg) beads was prepared using the calcium-hardening process, in brief, Ts-Car/Alg beads were prepared by combining sodium alginate (1.5%) and Ts-Car (1%) solutions. Dissolve 2 g Ts-Car in 100 mL distilled water for 30 min, stirring continuously. Then, distilled water (100 mL) and Na-alginate (3 g) were added separately then mixed for 30 min at room temperature until mixture became homogenous to get a viscous solution. Also, by adding the viscous solution (through a glass syringe) to a 3% CaCl_2_ solution while gradually stirring for hardening, spherical Ts-Car/Alg beads were created. The resulting beads were then rinsed by bi-distilled water to get rid of the calcium ions that had not yet reacted (Fig. 1).

#### Characterization

SEM was used at 15 kV beam energy. After surface dehydration in a desiccator for 2  h to evaluate the morphological surface of the synthesized Ts-Car/Alg and alginate hydrogel beads (scanning electron microscopy, Phenom, China). was used. Energy-dispersive X-ray spectroscopy (EDX) was employed to analyze the elemental makeup of the material utilized in the current study. Fourier transform infrared spectroscopy (FT-IR, NEXUS-670, USA) was utilized to analyze and confirm the modification in structure and functional groups of the prepared beads in a dry form after grinding them at room temperature. The powdered samples were measured in the range of 4000–500 cm^−1^ with resolution of 2 cm^−1^ at 16 scans per spectrum. A D-MAX 2500/PC (Japan Rigaku) X-ray diffractometer was used to record the X-ray diffraction (XRD) patterns of Ts-Car and Ts-Car/Alg beads to examine the crystalline structure. The instrument was operated in a continuous mode, and scanned over 2θ range of 10 to 90°.

#### Adsorption experiments

In this study, the Ts-Car/Alg gel beads were utilized to remove Pb^2+^ ions from their solutions. To evaluate the performance and potency of Ts-Car/Alg beads for adsorbing Pb^2+^ ions. Various adsorption parameters were investigated, including; (i) contact time effect which was conducted in conical flasks for (5, 15, 30, 60, and 120 min) using adsorbent beads (0.15 g) and100 mL Pb^2+^ ions solution (50 mg/L) at pH 5.3, (ii) influence of solution pH was examined over range (3–11) using 0.3 g Ts-Car/Alg gel beads and Pb^2+^ ions (100 mL) of (50 mg/L) for 120 min., where the pH of solution was adjusted by diluted NaOH (0.1N) and HCl (0.1N) solutions. (iii) The adsorbent dose effect was conducted in conical flasks with Ts-Car/Alg gel beads (0.05, 0.1, 0.2, 0.3, 0.4, and 0.5 g) and 100 mL of Pb^2+^ ions (50 mg/L) for 120 min at pH 5.3. (iv) Initial concentrations of Pb^2+^ ions were evaluated in the solution at different concentrations (10, 25, 50, 100, and 250 mg/L) over the course of 120 min at pH 5.3 and with 0.3 g of Ts-Car/Alg gel beads.

To maintain the ideal conditions for the hydrogels to achieve the best Pb^2+^ ions removal, all these various parameters that affect the adsorption process have been studied in a continuous agitation shaker at room temperature. Adsorbent beads were removed from the solution when the adsorption reaction was complete, and the amount of Pb^2+^ ions in the mixture was measured using atomic absorption spectrophotometry (Varian SpectrAA 220). The formula used to calculate the removal effectiveness and adsorption capacity is as follows:

The following formula was used to determine the adsorbent's removal efficiency:

1 $$R \%=\frac{\mathrm{Co }-\mathrm{ Ct }}{{\text{Co}}}\times 100$$

The capacity of adsorption, q (mg/g), was determined from:

2 $$q=\left(Co-Ct\right) \times \frac{V}{M}$$

where C_0_ and C_t_ are the initial and final concentrations of Pb^2+^ ion solutions (mg/L) after time (t), respectively, V is the volume of Pb^2+^ ions solution (mL) and M is Ts-Car/Alg gel beads (g) mass.

#### Adsorption kinetics

There exist two distinct models for adsorption kinetics, namely the pseudo-first-order and pseudo-second-order models. The metal adsorption process rate is explained using these models [31, 32].

To express the linear and non-linear pseudo-first-order use the following equations respectively, (3, 4):

3 $$\mathrm{log }\left({q}_{e}-{q}_{t}\right)=log{q}_{e}-\frac{{k}_{1}}{2.303}t$$

4 $${q}_{t}={q}_{e}-{e}^{-{k}_{t}t}$$

To express the linear and non-linear pseudo-first-order use the following equations respectively, (5, 6):

5 $$\frac{t}{{q}_{t}}=\frac{1}{{K}_{2}{q2}_{e}}+\left(\frac{1}{{q}_{e}}\right)t$$

6 $${q}_{t}=\frac{{k}_{2}{q}_{e}^{2}t}{1+{k}_{t}{q}_{e}t}$$

where, q_e_ (mg/g) and q_t_ (mg/g) are adsorption capacities of Ts-Car/Alg gel beads at equilibrium at time t (min), respectively, and ***k***_***1***_ (1/min) and ***k***_***2***_ [g/(mg min)] are the constants of first-order and second-order rate, respectively.

The following expression represented the adsorption rate h (mg/g min):

7 $$h={k}_{2}{q}_{e}^{2}$$

#### Adsorption isotherms

Adsorption isotherms were applied to show how metal ions interact with the synthesized adsorbents. Freundlich and Langmuir models are also used to examine adsorption processes [33].

Both the linear and non-linear Langmuir isotherms are defined using equations [8, 9], respectively:

8 $$qt/qmax =bCt/ (1+bCt)$$

9 $${q}_{t}=\frac{b{C}_{t}}{1+{q}_{m}{C}_{t}}$$

Both the linear and non-linear Freundlich isotherms are defined using equations [10, 11], respectively:

10 $$\mathit{ln}{q}_{e}=\mathit{ln}{k}_{f}+\left(\frac{1}{n}\right)\mathit{ln}{C}_{e}$$

where,* C*_*e*_ is the equilibrium concentration of Pb^2+^ ions (mg/L), *q*_*e*_ is the equilibrium sorption capacity (mg/g), *q*_*max*_ is the maximum sorption capacity (mg/g), and *b* is Langmuir constant (L/mg), which correlates to the adsorption energy and (*k*_*f*_ and *n*) are Freundlich isotherm constants.

The essential features of Langmuir isotherm can be described in terms of separation factor R_L_ which is described as:

11 $$R_{L = } {\raise0.7ex\hbox{$1$} \!\mathord{\left/ {\vphantom {1 {\left( {1 + bC_{O} } \right)}}}\right.\kern-0pt} \!\lower0.7ex\hbox{${\left( {1 + bC_{O} } \right)}$}}$$

where, R_L_ > 1 corresponds to unfavorable adsorption, R_L_ = 1 represents linear adsorption, R_L_ = 0 translates into irreversible, whereas R_L_ values between 0 and 1 indicate favorable adsorption.

#### Desorption and regeneration studies

An important study for lowering the overall cost of adsorbents is the desorption and regeneration of an absorbent. Adsorption–desorption was used to evaluate whether the produced gel beads for Pb^2+^ ions adsorption could be reused. 100 mL of pH 5.3 Pb^2+^ ions (50 mg/L) solution and 0.2 g of Ts-Car/Alg beads were weighed, added, and shaken for 1.5 h at room temperature. The Pb^2+^-loaded composite beads were taken out of the batch adsorption once the adsorption process was complete.

The remaining concentration of metal ions in the filtrate was then calculated. Afterwards, to desorb Pb^2+^ ions, 100 mL of 0.25 M HCl (the desorption medium) was added to the beads. The beads were then shaken for 1.5 h at room temperature. After being removed from the desorption medium, the beads were rinsed with distilled water until the pH was neutral, and they were then re-immersed in a solution containing 100 mL of Pb^2+^ ions (50 mg/L) for 1.5 h to begin the subsequent adsorption cycle. This experiment was repeated twice, and the succeeding adsorption procedure used the previously used adsorbent.

## Results and discussion

### Characterization

**Fig. 2:**
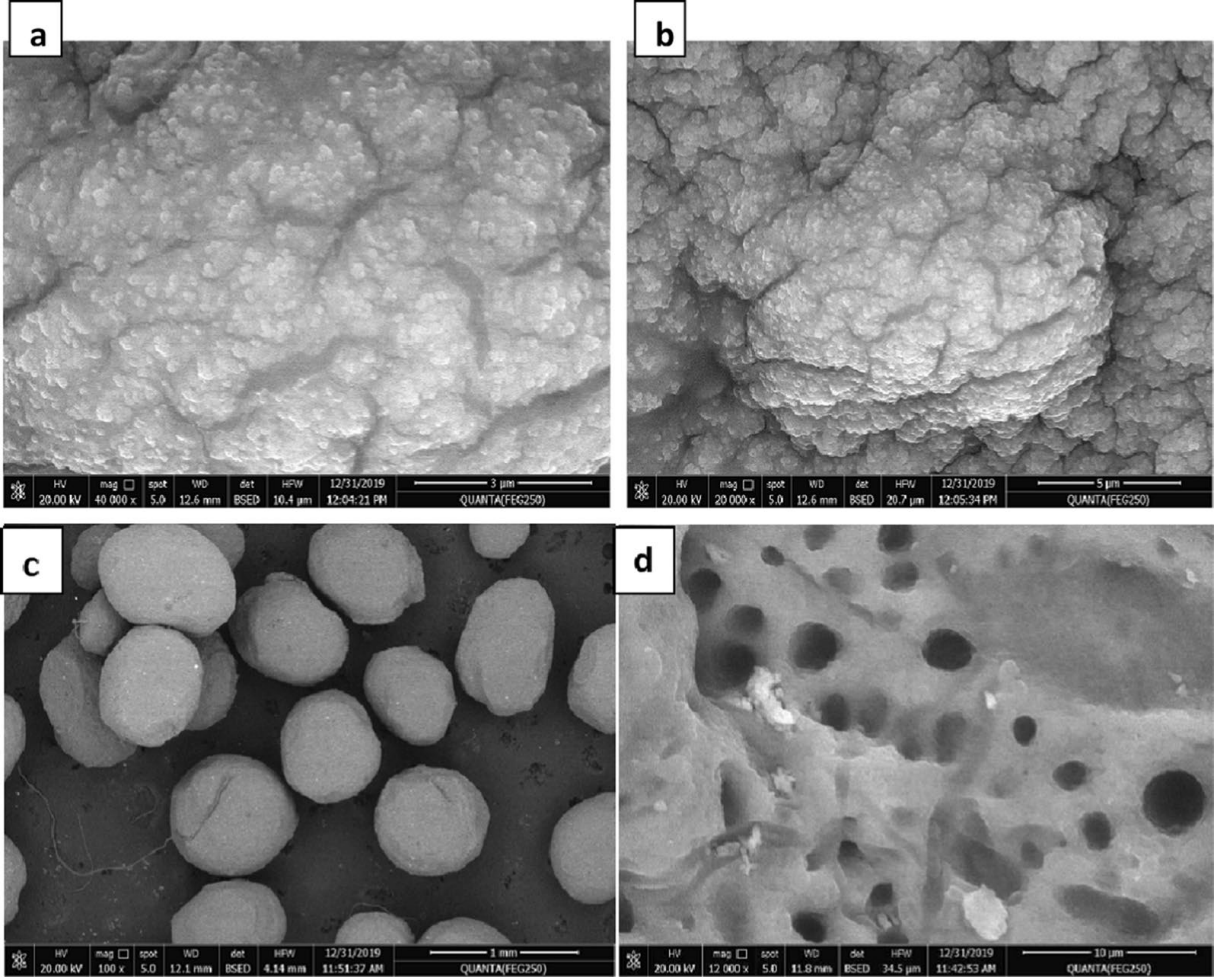
SEM images of alginate beads (**a**, **b**) and Ts-Car/Alg beads (**c**, **d**)

Scanning electron microscopy was used to analyze the surface morphology and porosity development of alginate beads and Ts-Car/Alg beads (SEM). As shown in Fig. 2a, b the alginate beads' surface is smooth and devoid of any grooves, indicating that there is no porosity there. In contrast to the surface of Ts-Car/Alg beads, as shown in Fig. 2c, d, is full of grooves due to crosslinking with Ts-car, which provided a high surface area and large numbers of active sites to accommodate metal ions, enhancing Pb^2+^ ion adsorption efficiency. Given that their pores can operate as active sites for the adsorption of metal ions, these beads are viable candidates for water treatment, according to the surface morphology.

**Fig. 3:**
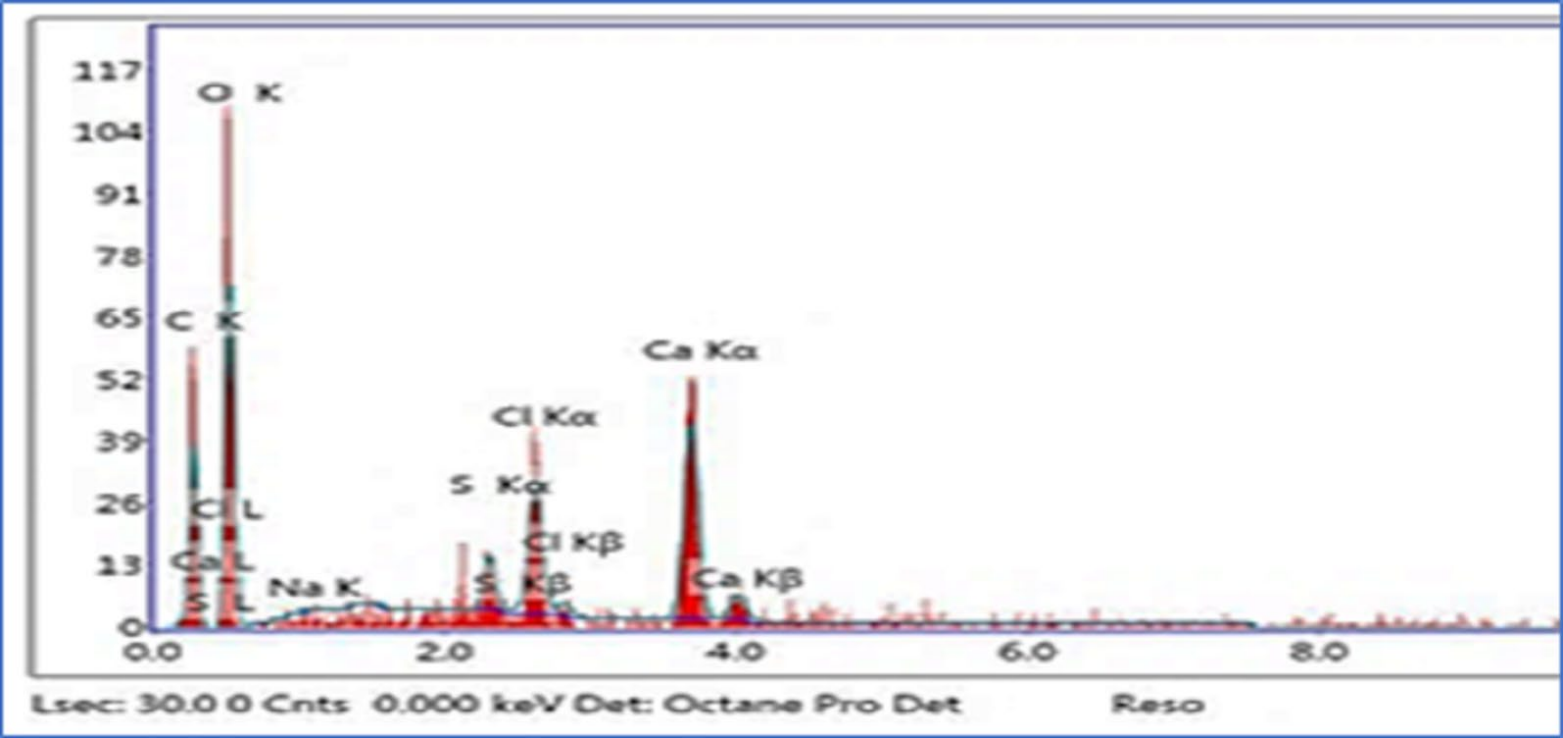
EDX patterns of Ts-Car/Alg beads

Figure 3 is the EDX pattern of Ts-Car/Alg gel beads that displayed the distribution of the appropriate elements of the produced matrix. The presence of just C, O, S (belongs to Ts), S, O (belongs to carrageenan), C, O (belongs to Alginate), and Ca (belongs to a cross-linking agent) in the spectrum revealed a highly pure Ts-Car/Alg matrix.

**Fig. 4:**
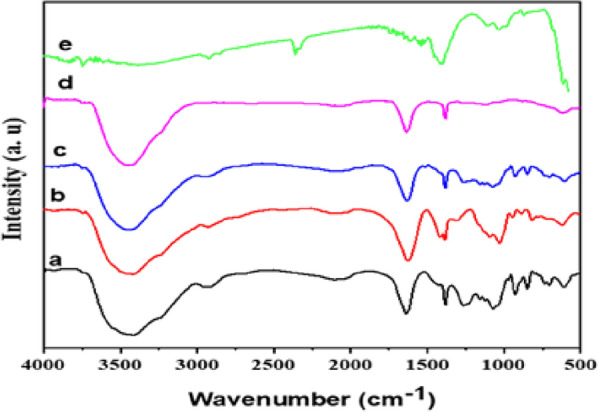
**a** FTIR spectra of Car., **b** NaAlg, **c**Ts-Car., **d** Ts-Car/Alg, and **e** Ts-Car/Alg (after adsorption) Beads

FTIR spectra analysis was used to elucidate the development of the new matrices. Carrageenan FTIR analysis, as seen in Fig. 4a showed stretching vibration of C_4_–O–S and a symmetric vibration of O=S=O at 1265 cm^−1^ and 848 cm^−1^, respectively. The peaks at 925 cm^−1^ revealed 3,6-anhydrous-galactose contains C–O–C. Moreover, O–H stretching, C–H stretching, C–O stretching, and C–O stretching, respectively, were associated with bands at 3417 cm^−1^, 2908 cm^−1^, 1157 cm^−1^, and 1072 cm^−1^. The O–H bending has a characteristic peak at 1643 cm^−1^. Furthermore, the stretching vibration band of S=O in the (SO_4_) group is referenced by the peak in the spectrum of Car. at 1261 cm^−1^ [34–36]. Figure 4b of the FTIR of sodium alginate showed significant bands of the hydroxyl, ether, and carboxylic functional groups. O–H stretching vibrations appeared at 3417 cm^−1^, while carboxylate O–C–O asymmetric stretching vibrations has two other bands seen at 2931 and 1620 cm^−1^, respectively. Additionally, the O–C–O symmetric stretching vibration of the carboxylate group contributed to the absorption band at 1419 cm^−1^, which was related to the C–OH deformation vibration. In addition, the pyranose rings’ C–C–H (and O–C–H) deformation, C–O stretching vibrations, and C–O (and C–C) stretching vibrations, represent the bands at 1388, 1095, and 1033 cm^−1^, respectively. Furthermore, the peak at 948 cm^−1^ coincided with the presence of uronic acid as indicated by the C–O stretching vibration. The peaks at 887 and 817 cm^−1^ were due to the mannuronic acid residues and the l-gulopyranuronic asymmetric ring vibration, respectively [37–39]. By comparing Fig. 4c and d, the FT-IR spectra shows bands that Ts-Car/Alg at 3448 cm^−1^, OH groups first emerged, and at 2962 cm^−1^, C–H stretching began. The band for C–O–C was visible at 1072 cm^−1^, while other bands that are typical for tosylate groups were seen at 1126 cm^−1^ for SO_2_, 1381 cm^−1^ for S–O, 2973 cm^−1^ for C–H aromatic, and 1635 cm^−1^ for C–C aromatic, respectively [40, 41].

The FTIR chart of the beads after adsorption (Fig. 4e) supported the adsorption mechanism. The decrease in the intensity of the carboxylate group band of O–C–O symmetric stretching vibration at 1419 cm^−1^ and the band of OH groups at 3448 cm^−1^ revealed their contribution in the adsorption of lead ions. The result of the FTIR also highlights the participation of both the carboxylate and hydroxyl groups in bonding with lead ions.

**Fig. 5:**
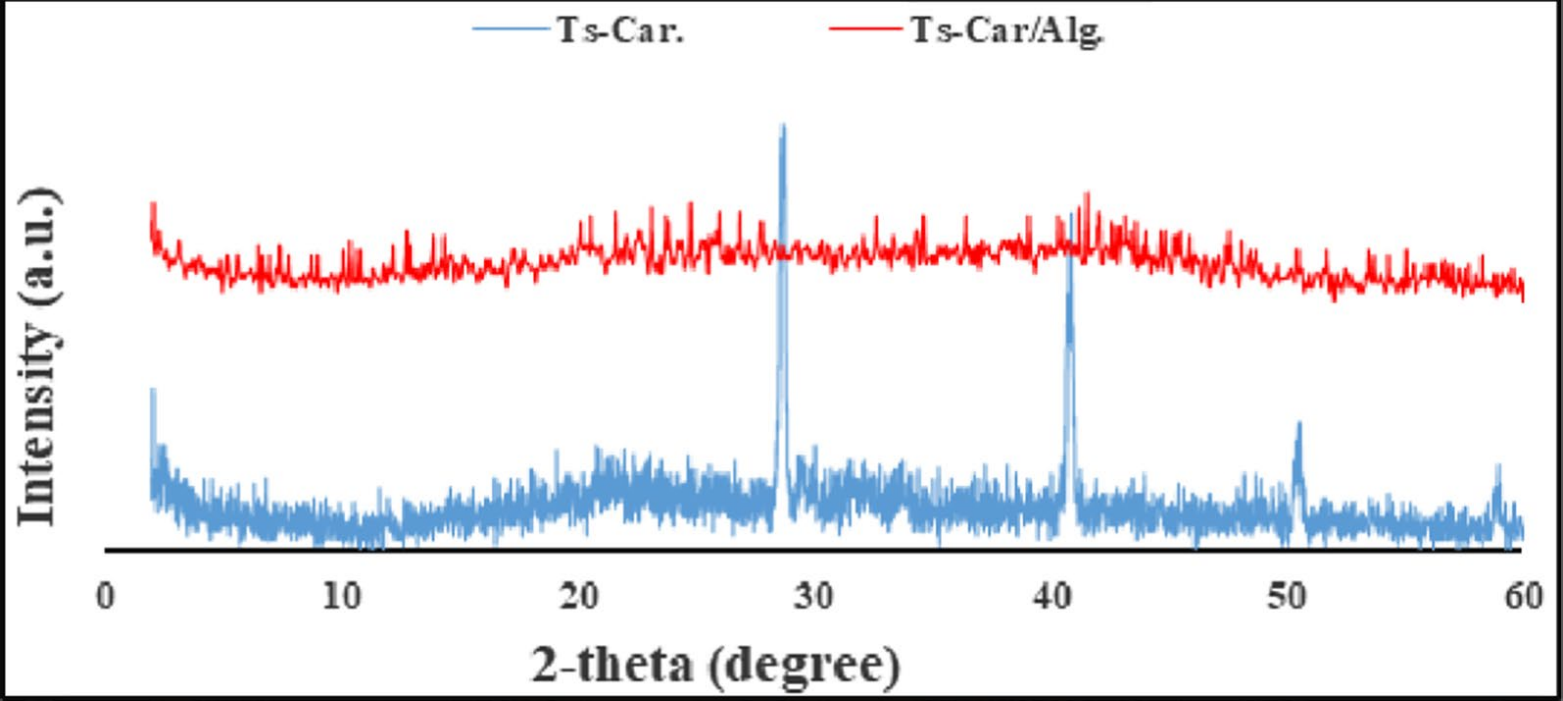
XRD pattern of Ts-Car. & Ts-Car/Alg Beads

The results of an XRD analysis of Ts-Car and Ts-Car/Alg beads are shown in Fig. 5. The first weak band and the second powerful peak, which are typically detected at 2θ about 20° and 28°, respectively, are attributed to the carrageenan base. While the alginate is thought to be responsible for the typical peaks at 2θ about 40° and 50°. Given that crosslinking has led to lower crystallinity and impair chain mobility, the combination of Ts-Carrageenan and Alginate significantly inhibited the crystallinity of the peaks of Ts-Carrageenan [42].

### Adsorption study

#### Contact time effect on Pb^2+^ ions adsorption.

It is known that contact time plays a crucial role in determining how metal ions are eliminated when examining the adsorption kinetics of an adsorbent. The impact of contact duration on Pb^2+^ ions removal by Ts-Car/Alg beads was examined. By adding 0.15 g of Ts-Car/Alg beads to a solution of 50 mg/L Pb^2+^ ions and a pH of 5.3, with keeping all other variables constant, the mixture was agitated and samples were taken every 5, 15, 30, 60, and 120 min at room temperature.

**Fig. 6:**
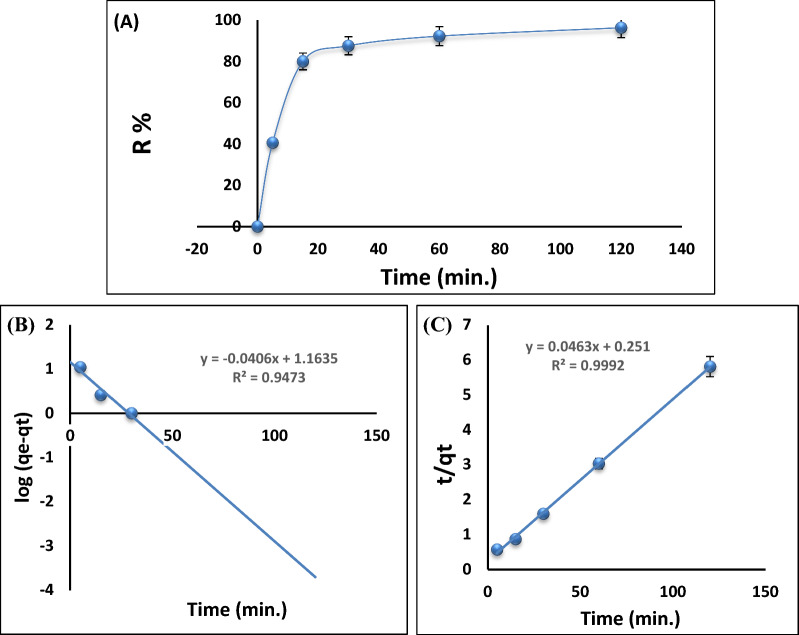
Contact time influence on the Pb^2+^ ions adsorption (**A**), linear Pseudo-first-order (**B**), and linear Pseudo-second-order (**C**) for the adsorption of 50 (mg/L) Pb^2+^ ions onto 0.15 g Ts-Car/Alg beads

At each time, the amount of Pb^2+^ ions left in the solution was calculated (Fig. 6). According to the results, the amount of Pb^2+^ ions adsorbed onto Ts-Car/Alg beads noticeably increases from 40.62 to 96.25% with the increase in contact duration till reaching equilibrium after 60 min. This is mostly due to the considerable number of active sites that are present on the adsorbent surface, which were later occupied with metal ions [43]. Pseudo-first order and pseudo-second-order kinetic models have been studied to better understand the kinetics of Pb^2+^ ions being adsorbed by Ts-Car/Alg beads. The kinetic model's constants and correlation coefficients, which were calculated and listed in Table 1, showed that the adsorption reaction's kinetics follows pseudo-second-order model (Fig. 6), as evidenced by the higher correlation coefficient (R^2^ = 0.9992) value and the similar value between the calculated and experimental qe, which simulate that the sorption rate is proportionate to the square of the number of unoccupied binding sites [44].

**Table 1:** linear Kinetics constants for Pb^2+^ ions adsorption by Ts-Car/Alg beads

Pseudo-first-order	k_1_ (min^−1^)	q_e_ (exp.) (mg/g)	q_e_ (cal.) (mg/g)	R^2*^	SSE^**^
	0.093	20.63	14.57	0.9473	15.4

Pseudo-second-order	K_2_ (g/mg min)	q_e_ (exp.) (mg/g)	q_e_ (cal.) (mg/g)	H (mg/g min)	R^2^	SSE
	0.0085	20.63	21.6	3.96	0.9992	0.015

^*^R^2^: The regression coefficient factor

^*^SSE: The Sum of Squared Errors

#### Effect of pH on Pb^2+^ ions adsorption

**Fig. 7:**
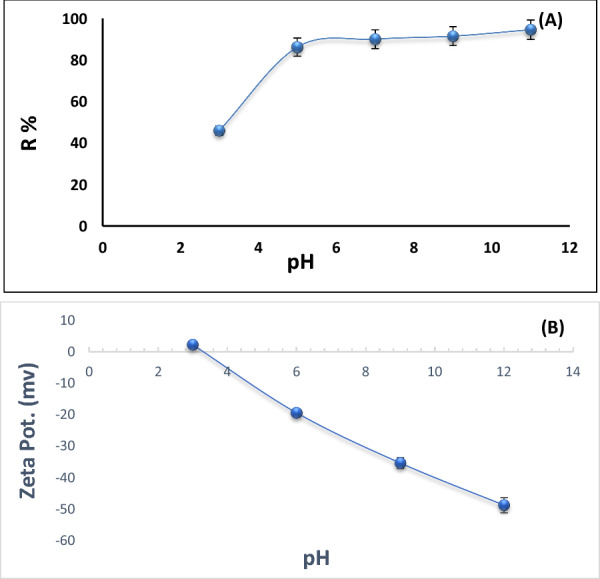
**a** pH Effect on the adsorption [0.15 g adsorbent dosage, 100 mL Pb^2+^ ions; 50 mg/L, and contact time 120 min, room temperature]. **b** Determination of zero-point charge

In aqueous solutions, the ionization of functional groups that are present onto the surface of the adsorbent (charges of adsorption sites) is significantly influenced by pH, which is a characteristic parameter that impacts the behavior of the adsorption of heavy metal ions [45]. Experiments were conducted with 100 mL Pb^2+^ ions solution (50 mg/L) containing 0.15 g Ts-Car/Alg beads in varied solution pH range 3–11 at room temperature for 2 h, while other variables were held constant. The results are shown in Fig. 7, which shows that as the pH rises, the effectiveness of removing Pb^2+^ ions increase from 46 to 94.5%. Hydrogen protons (H^+^) in the medium and Pb^2+^ ions competed (via electrostatic repulsion) for the same binding sites at low pH, which reduced adsorption capability. However, the removal percentage rose when the pH of the solution was raised because the competition between the Pb^2+^ ions and hydrogen protons (H^+^) for adsorption sites was eliminated. However, when the pH is more than 8, the Pb^2+^ ions started to precipitate as Pb (OH)_2_ hydroxides, which is crucial for the elimination of Pb^2+^ ions [46]. So the optimal pH range for effective Pb^2+^ ions adsorption lies between 5 and 6 to prevent Pb^2+^ ions precipitation, as precipitation could potentially undermine the overall efficacy of the adsorption process. The pH points zero charge (pH _ZPC_) was determined using Zeta potentials (Zetasizer Nano S, Malvern Instruments, UK) at different pH (3–12) and revealed that pH _ZPC_ is 3.2. It is clear from Fig. 7b that a lower pH value than pH_ZPC_ leads to a higher density of positive ions on the surface of the beads, which in turn allows less adsorption. When the pH of the solution is higher than pH_ZPC,_ a negative charge is present on the surface of the beads, which leads to better adsorption of lead cations through the phenomenon of electrostatic attraction.

#### Effect of adsorbent dosage on Pb^2+^ ions adsorption

The dosage of the adsorbent is one of the factors that is known to have the biggest influence on the adsorption process. Using different amounts of Ts-Car/Alg beads (0.05, 0.1, 0.2, 0.3, 0.4, 0.5) on Pb^2+^ ions uptake (Pb^2+^ ions solution 50 mg/L, at pH 5.3), while maintaining all other parameters constant, the effect of adsorbent dosage on Pb^2+^ ions elimination from aqueous solutions was investigated. The prepared solutions were then shaken for 120 min. at room temperature.

**Fig. 8:**
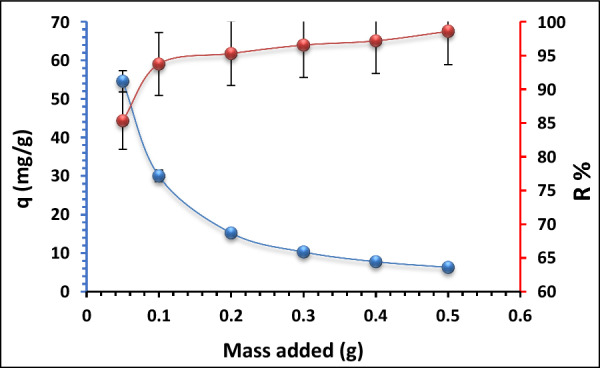
Influence of adsorbent dosage on adsorption of Pb^2+^ ions (C_0_ = 50 mg/L, pH 5.3, contact time 120 min, and room temperature)

Figure 8 revealed that when the Ts-Car/Alg bead dose rose from 0.05 to 0.5 gm/L, the adsorption capacity declined from 54.6 to 6.31 mg/g. This is because more adsorbent sites were added to the solution, which caused them to compete with one another and reduce the adsorption capacity. The removal efficacy rose from (85.3 to 98.6%) as the adsorbent dosage was raised from 0.05 to 0.5 gm/L. This is because the surface of the adsorbent had more active sites available for the adsorption of Pb^2+^ ions [47, 48]. Therefore, the optimal sorbent dosage is determined to be 0.3 g once equilibrium is attained.

#### Effect of initial Pb^2+^ ions concentration

**Fig. 9:**
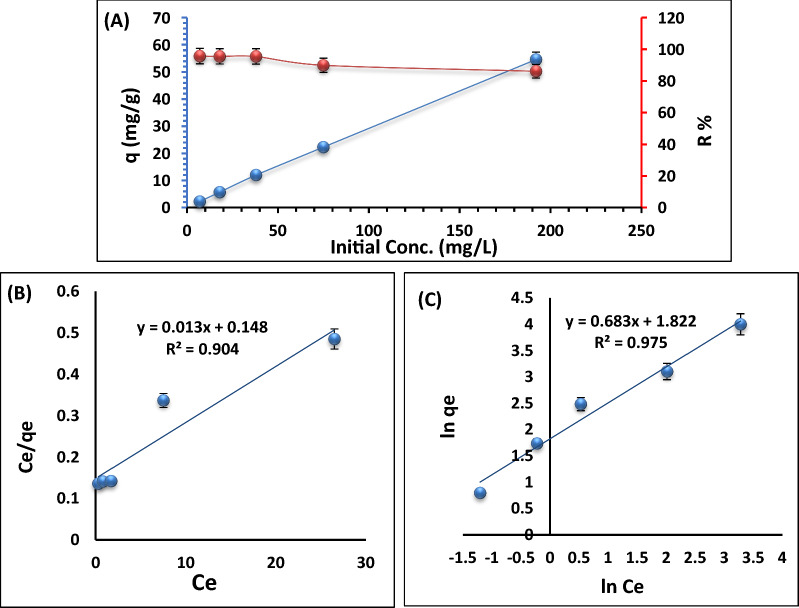
Influence of initial Pb^2+^ ions concentration on the removal effectiveness and uptake capacity of 0.15 g Ts-Car/Alg beads (**A**), linear Langmuir (**B**), and linear Freundlich (**C**) adsorption isotherm models of Pb^2+^ ions adsorption onto Ts-Car/Alg beads

The amount of Pb^2+^ ions present at the beginning has a significant effect on the adsorption process over time. Therefore, at room temperature, solutions containing 10, 25, 50, 100, and 250 mg/L of Pb^2+^ ions were shaken with 0.3 g of Ts-Car/Alg beads. From the results displayed in Fig. 9a, it was clear that at lower Pb^2+^ ion concentrations (50 mg/L), the removal rate rose (R% 95.7%), whereas at higher concentrations (> 50 mg/L), the removal rate was declined (R% 86.2%). The rise in Pb^2+^ ions removal rate at low initial Pb^2+^ ions concentration is caused by the adsorbent surface having enough open active sites. As a result of a lack of active sites on the adsorbent, the adsorption rate slowed down with increasing initial Pb^2+^ ions concentration [49].

On the other hand, the adsorption capacity rose from 2.2 to 54.6 mg/g with the rise in Pb^2+^ ions concentration, this was attributed to the ratio between Pb^2+^ and Ts-Car/Alg beads was enough to afford a driving force to overcome the mass transport resistance between them [50]. To describe the adsorption process more clearly between Ts-Car/Alg beads adsorbent and Pb^2+^ ions, both Freundlich isotherm and Langmuir models have been examined (Fig. 9b).

The study of the constants and correlation coefficients of the isotherm model is reported in Table 2. The greater correlation coefficient value (R^2^ = 0.975) indicates that Pb^2+^ ions were adsorbed onto the Ts-Car/Alg beads’ surface in multiple layers. This finding supports the conclusion that the adsorption process adheres to the Freundlich isotherm (as shown in Fig. 9c), which posits the multilayer adsorption of lead ions onto Ts-Car/Alg beads [47]. Furthermore, since the n value in Freundlich isotherm was higher than one, the process of adsorption was physically accomplished. Langmuir isotherm RL value, which ranges from 0 to 1, was get to be in this range, indicating that the adsorption of Pb^2+^ ions onto Ts-Car/Alg beads was successfully processed. The results reveal that Ts-Car/Alg beads displayed a remarkable Pb^2+^ uptake capacity (qmax) of 74 mg/g, surpassing the capacities of previously reported sorbents, as demonstrated in Table 3.

**Table 2:** linear Freundlich and linear Langmuir isotherms constants of Pb^2+^ ions adsorption onto Ts-Car/Alg beads adsorbent

Freundlich Constants				Langmuir Constants			
K_f_	n	R^2^	SSE	q_m_ (mg/g)	b (L/mg)	R^2^	SSE
6.2	1.46	0.9755	0.1487	74	0.09	0.9045	0.0095

**Table 3:** A comparative study of Pb^2+^ adsorption with similar composite materials

Sorbents	Adsorption capacity, (mg/g)	References
Modified beech sawdust	2.46	[51]
Activated carbon and Chemically-modified activated carbon	30.7 30.6	[52]
Melamine- pyridine polyaminal network (MA-Py)	53.13	[53]
Ts-Car/Alg beads	74 mg/g	Present work

#### Regeneration

One crucial step in the process of water treatment is the regeneration of the absorbent [54]. Two cycles of regeneration research were completed. The results showed that the first cycle Pb^2+^ ion removal effectiveness exceeded 95.3% before dropping to 80% in the second cycle. It is important to note that Ts-Car/Alg beads can be used more than twice to remove heavy metal ions from contaminated water.

#### Adsorption mechanism

**Fig. 10:**
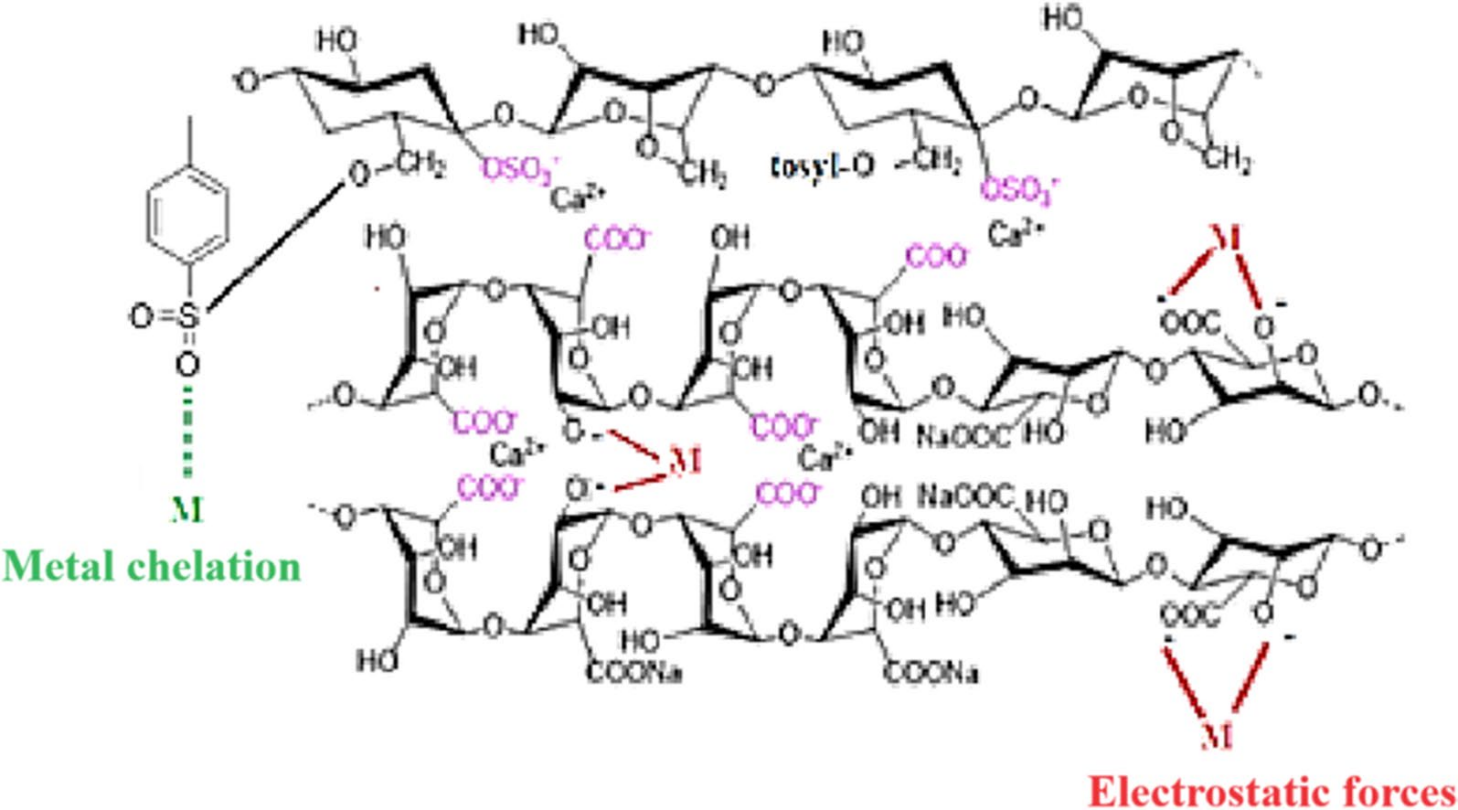
Schematic diagram of adsorption mechanism of Tosyl-carrageenan/Alginate (Ts-Car/Alg) beads for Pb^2+^ ions

Figure 10 revealed that a contact took place between Pb^2+^ ions and Ts-Car/Alg beads. The locations where metal ions were attached to Ts-Car/Alg beads that contain available negatively charged functional groups (–COO^−^, –OSO_3_^−^, –SO_2_^−^…) that were bonded to positively charged Pb^2+^ ions through electrostatic attraction and chelation, which were confirmed, in brief, using a variety of characterization methods.

## Conclusions

The prepared Ts-Car/Alg beads are promising for removing Pb^2+^ ions from aqueous solutions. Their remarkable adsorption capacity, coupled with their ease of use, positions them as a valuable resource for addressing heavy metal ion pollution. We have diligently confirmed their formation and effectiveness through rigorous material characterization and extensive adsorption studies. Our findings indicate that the adsorption equilibrium increases as the concentration of Pb^2+^ ions rise, particularly at a pH of 5.3, after a contact time of 120 min, and 0.3 g of Ts-Car/Alg demonstrated the highest adsorption capacity at 74 mg/g. furthermore, its successful regeneration and reusability evaluation for water treatment, make them a noteworthy addition to the field of adsorbent materials.

## Data Availability

The Data and materials for this paper are available with the corresponding author upon request. [Dr Korany A. Ali email: *ka.khalil@nrc.sci.eg*, *kornykhlil@gmail.com*].

## References

[1] Abdolmaleki A, Mallakpour S, Borandeh S (2015). Efficient heavy metal ion removal by triazinyl-bcyclodextrin functionalized iron nanoparticles. RSC Adv.

[2] Tang S, Yang J, Lin L, Peng K, Chen Y, Jin S, Yao W (2020). Construction of physically crosslinked chitosan/sodium alginate/calcium ion double-network hydrogel and its application to heavy metal ions removal. Chem Eng J.

[3] Wu Z, Deng W, Zhou W, Luo J (2019). Novel magnetic polysaccharide/graphene oxide @Fe3O4 gel beads for adsorbing heavy metal ions. Carbohyd Polym.

[4] Lai KC, Lee LY, Hiew BYZ, Gopakumar ST, Gan S (2020). Facile synthesis of xanthan biopolymer integrated 3D hierarchical graphene oxide/titanium dioxide composite for adsorptive lead removal in wastewater. Biores Technol.

[5] Bahrami M, Amiri MJ, Bagheri F (2019). Optimization of the lead removal from aqueous solution using two starch-based adsorbents: design of experiments using response surface methodology (RSM). J Environ Chem Eng.

[6] Zhuang S, Zhang Q, Wang J (2021). Adsorption of Co^2+^ and Sr^2+^ from aqueous solution by chitosan grafted with EDTA. J Mol Liq.

[7] Mousa NE, Simonescu CM, Pătescu RE, Onose C, Tardei C, Culiţă DC, Oprea O, Patroi D, Lavric V (2016). Pb^2+^ removal from aqueous synthetic solutions by calcium alginate and chitosan coated calcium alginate. React Funct Polym.

[8] Giri TK, Verma D, Tripathi DK (2015). Effect of adsorption parameters on biosorption of Pb^++^ ions from aqueous solution by poly (acrylamide)-grafted kappa-carrageenan. Polym Bull.

[9] Eskhan A, Banat F, Haija MA, Al-Asheh S (2019). Synthesis of mesoporous/macroporous microparticles using threedimensional assembly of chitosan-functionalized halloysite nanotubes and their performance in the adsorptive removal of oil droplets from water. Langmuir.

[10] Vázquez OFG, del Virgen MRM, Montoya VH, Gómez RT, Flores JLA, Perez-Cruz MA, Morán MÁM (2016). Adsorption of heavy metals in the presence of a magnetic field on adsorbents with different magnetic properties. Ind Eng Chem Res.

[11] Bertagnolli C, Grishin A, Guibal E, Vincent T (2015). Synthesis and application of a novel sorbent (tannic acid-grafted-polyethyleneimine encapsulated in alginate beads) for heavy metal removal. Sep Sci Technol.

[12] Jiang X, An Q-D, Xiao Z-Y, Zhai S-R, Shi Z (2019). Versatile core/shell-like alginate@polyethylenimine composites for efficient removal of multiple heavy metal ions (Pb^2+^, Cu^2+^, CrO_4_^2-^): batch and fixedbed studies. Mater Res Bull.

[13] Liu M, Wen Y, Song X, Zhu J-L, Li J (2019). A smart thermoresponsive adsorption system for efficient copper ion removal based on alginate-g-poly (n-isopropylacrylamide) graft copolymer. Carbohyd Polym.

[14] Guo D-M, An Q-D, Xiao Z-Y, Zhai S-R, Yang D-J (2018). Efficient removal of Pb (II), Cr(VI) and organic dyes by polydopamine modified chitosan aerogels. Carbohyd Polym.

[15] Wang Y, Feng Y, Zhang X-F, Zhang X, Jiang J, Yao J (2018). Alginate-based attapulgite foams as efficient and recyclable adsorbents for the removal of heavy metals. J Colloid Interface Sci.

[16] Feng G, Ma J, Zhang X, Zhang Q, Xiao Y, Ma Q (2019). Wang S (2019) Magnetic natural composite Fe3O4-chitosan@bentonite for removal of heavy metals from acid mine drainage. J Colloid Interface Sci.

[17] Li S-S, Song Y-L, Yang H-R, An Q-D, Xiao Z-Y, Zhai S-R (2020). Modifying alginate beads using polycarboxyl component for enhanced metal ions removal. Int J Biol Macromol.

[18] Sutirman ZA, Sanagi MM, Aini WIW (2021). Alginate-based adsorbents for removal of metal ions and radionuclides from aqueous solutions: a review. Int J Biol Macromol.

[19] Gao C, Wang X-L, An Q-D, Xiao Z-Y, Zhai S-R (2021). Synergistic preparation of modified alginate aerogel with melamine/ chitosan for efficiently selective adsorption of lead ions. Carbohyd Polym.

[20] Thakur S (2021). An overview on alginate-based bio-composite materials for wastewater remedial. Mater Today Proc.

[21] Kulal P, Badalamoole V (2020). Hybrid nanocomposite of kappa-carrageenan and magnetite as adsorbent material for water purification. Int J Biol Macromol.

[22] Abdullah S, Azeman NH, Mobarak NN, Zan MSD, Bakar AAA (2018). Sensitivity enhancement of localized SPR sensor towards Pb (II) ion detection using natural biopolymer-based carrageenan. Optik.

[23] Narayanan KB, Han SS (2017). Highly selective and quantitative colorimetric detection of mercury (II) ions by carrageenan-functionalized Ag/AgCl nanoparticles. Carbohydr Polym.

[24] Abou-Zeid RE, Ali KA, Gawad RMA, Kamal KH, Kamel S, Khiari R (2021). Removal of Cu (II), Pb(II), Mg(II), and Fe(II) by adsorption onto alginate/nanocellulose beads as bio-sorbent. J Renew Mater.

[25] Ali KA, Wahba MI, Abou-Zeid RE, Kamel S (2019). Development of carrageenan modified with nanocellulose-based materials in removing of Cu^2+^, Pb^2+^, Ca^2+^, Mg^2+,^ and Fe2+. Int J Environ Sci Technol.

[26] Iqbal M, Saeed A (2009). Zafar SI (2009) FTIR spectrophotometry, kinetics and adsorption isotherms modeling, ion exchange, and EDX analysis for understanding the mechanism of Cd^(2+)^ and Pb^(2+)^ removal by mango peel waste. J Hazard Mater.

[27] Gerickea M, Heinzea T (2015). Homogeneous tosylation of agarose as an approach toward novel functional polysaccharide materials. Carbohyd Polym.

[28] Bretschneider L, Koschella A, Heinze T (2015). Cationically modified 6-deoxy-6-azido cellulose as a water-soluble and reactive biopolymer derivative. Polym Bull.

[29] Yu F, Cui T, Yang C, Dai X, Ma J (2019). k-Carrageenan/Sodium alginate double-network hydrogel with enhanced mechanical properties, anti-swelling, and adsorption capacity. Chemosphere.

[30] Yang P, Yu F, Yang Z, Zhang X, Ma J (2022). Graphene oxide modified κ-carrageenan/sodium alginate double-network hydrogel for effective adsorption of antibiotics in a batch and fixed-bed column system. Sci Total Environ.

[31] Ho YS, Mckay G (1998). A comparison of chemisorption kinetic models applied to pollutant removal on various sorbents. Trans IChemE.

[32] Ho YS, McKay G (1999). Pseudo-second order model for sorption processes. Process Biochem.

[33] Langmuir The (1916). constitution and fundamental properties of solids and liquids. Part 1. Solids. J Am Chem Soc.

[34] Covis R, Guegan J-P, Jeftić J, Czjzek M, Benoit M, Benvegnu T (2016). Structural and rheological properties of kappa (κ)-carrageenans covalently modified with cationic moieties. J Polym Res.

[35] Vanegas JS, Torres GR, Campos BB (2019). Characterization of a κ-carrageenan hydrogel and its evaluation as a coating material for fertilizers. J Polym Environ.

[36] Rao KM, Rao KSVK, Sudhakar P, Rao KC, Subha MCS (2013). Synthesis and characterization of biodegradable poly (Vinyl caprolactam) grafted on to sodium alginate and its microgels for controlled release studies of an anticancer drug. J Appl Pharm Sci.

[37] Pereira R, Tojeira A, Vaz DC, Mendes A, Bártolo P (2011). Preparation and characterization of films based on alginate and aloe vera. Int J Polym Anal Charact.

[38] Li P, Dai Y-N, Zhang J-P, Wang A-Q, Wei Q (2008). Chitosan-alginate nanoparticles as a novel drug delivery system for nifedipine. Int J Biomed Sci.

[39] Kuczajowska-Zadrożna M, Filipkowska U, Jóźwiak T (2020). Adsorption of Cu (II) and Cd (II) from aqueous solutions by chitosan immobilized in alginate beads. J Environ Chem Eng.

[40] El Hamdaoui L, Talbaoui A, El Moussaouiti M (2021). Nucleophilic displacement reaction on tosyl cellulose by l-Methionine to the synthesis of novel water-soluble cellulose derivative and its antibacterial activity. Int J Polym Sci.

[41] Lambert WS, Phillips PJ (1994). Small-angle X-ray scattering studies of crystallization in crosslinked linear polyethylene. Polymer.

[42] Ravulapalli S, Kunta R (2018). Removal of lead (II) from wastewater using active carbon of *Caryota urens* seeds and its embedded calcium alginate beads as adsorbents. J Environ Chem Eng.

[43] Hassan MA, Mohammad AM, Salaheldin TA, El-Anadouli BE (2018). A promising hydroxyapatite/graphene hybrid nanocomposite for methylene blue dye’s removal in wastewater treatment. Int J Electrochem Sci.

[44] Ren H, Gao Z, Wu D, Jiang J, Sun Y, Luo C (2016). Efficient Pb(II) removal using sodium alginate-carboxymethyl cellulose gel beads: preparation, characterization, and adsorption mechanism. Carbohydr Polym.

[45] Dacrory S, Kamal KH, Kamel S (2021). EDTA-functionalized magnetic graphene oxide/polyacrylamide grafted carboxymethyl cellulose hydrogel for removal of Pb^+2^ from aqueous solution. J Polym Environ.

[46] Al-Sakkari EG, Abdeldayem OM, Genina EE, Amin L, Bahgat NT, Rene ER, El-Sherbiny IM (2020). New alginate-based interpenetrating polymer networks for water treatment: a response surface methodology based optimization study. Int J Biol Macromol.

[47] Wang H, Wang S, Chen Z, Zhou X, Wang J, Chen Z (2020). Engineered biochar with anisotropic layered double hydroxide nanosheets to simultaneously and efficiently capture Pb^2+^ and CrO_4_^2−^ from electroplating wastewater. Biores Technol.

[48] Liang R-H, Li Y, Huang L, Wang X-D, Hu X-X, Liu C-M, Chen M-S, Chen J (2020). Pb^2+^ adsorption by ethylenediamine-modified pectins and their adsorption mechanisms. Carbohydr Polym.

[49] Alamrani NA, Al-Aoh HA, Aljohani MMH, Bani-Atta SA, Sobhi M, Khalid MS, Darwish AAA, Keshk AA, Abdelfattah MAA (2021). Wastewater purification from permanganate ions by sorption on the *Ocimum basilicum* leaves powder modified by zinc chloride. J Chem.

[50] Oun AA, Kamal KH, Farroh K, Ali EF, Hassan MA (2021). Development of fast and high-efficiency sponge-gourd fibers (*Luffa cylindrica*)/hydroxyapatite composites for removal of lead and methylene blue. Arab J Chem.

[51] Tashauoei HR, Hashemi S, Ardani R, Yavari Z, Asadi-Ghalhari M (2016). Adsorption of lead from aqueous solution by modified beech sawdust. J Saf Environ Health Res.

[52] Abdulkarim M, Al-Rub FA (2004). Adsorption of lead ions from aqueous solution onto activated carbon and chemically-modified activated carbon prepared from date pits. Adsorpt Sci Technol.

[53] Sandín R, González-Lucas M, Sobarzo PA, Terraza CA, Maya EM (2021). Microwave-assisted melamine-based polyaminals and their application for metal cations adsorption. Eur Polymer J.

[54] Rivas BL, Villegas S, Ruf B, Peric IM (2007). Removal of metal ions with impact on the environment by water-insoluble functional copolymers: synthesis and metal ion uptake properties. J Chil Chem Soc.

